# Management of radiology services during the 2022 FIFA football (soccer) World Cup

**DOI:** 10.1007/s00256-023-04486-2

**Published:** 2023-11-09

**Authors:** Marcelo Bordalo, Toni Evans, Salwa Allenjawi, Stephen Targett, Peter Dzendrowskyj, Abdulaziz Jaham Al-Kuwari, Marco Cardinale, Pieter D’Hooghe

**Affiliations:** Aspetar Orthopedic and Sports Medicine Hospital, Doha, Qatar

**Keywords:** Management, Radiology, Musculoskeletal, FIFA, Football, Soccer, Radiology report, Audiovisual report

## Abstract

Diagnostic imaging is the predominant medical service sought for the assessment and staging of musculoskeletal injuries in professional sports events. During the 2022 FIFA Football (soccer) World Cup, a centralized medical care infrastructure was established. This article provides a comprehensive account of the radiological services implemented during this event, encompassing the deployment of equipment and human resources, the structuring of workflows to uphold athlete confidentiality, and initiatives aimed at enhancing communication. Communication channels were refined through radiology consultations held with national teams’ health care providers and the adoption of audiovisual reports available in multiple languages, which could be accessed remotely by team physicians. Our established framework can be replicated in international professional football events for seamless integration and efficacy.

**Supplementary Information:**

The online version contains supplementary material available at 10.1007/s00256-023-04486-2.

## Introduction

The FIFA (Fédération Internationale de Football Association) football (or soccer) World Cup is the largest and most widely watched single-sport competition in the world, with close to 1.5 billion people watching the final match [1]. Typically, the tournament is hosted across multiple cities with considerable distances between venues. However, the 2022 World Cup took place in Qatar, where all the stadiums were concentrated within a 55-km radius of the capital city of Doha. Consequently, a centralized approach to athlete medical care was possible.

Managing team medical services at a major sports event poses a considerable challenge for health care professionals. Over the years, the volume of imaging studies at such events has seen a substantial increase, possibly due to the accessibility of services and subsequent improvements in technology and speed of imaging, as well as the need to make quick decisions about athletes’ return to play and replacement [2–5]. Despite the multitude of reports on large multisports events, there is a scarcity of literature documenting radiology management and organizational necessities specific to major football tournaments.

In this article, we describe the strategic planning and execution of radiological services during the 2022 FIFA World Cup held in Qatar.

## Scope of medical care

Aspetar Orthopedic and Sports Medicine Hospital was appointed as the primary medical care provider for the 32 national teams who took part in the competition, with a cohort comprising 832 athletes, an estimated 1300 team members, FIFA staff, 130 referees, and an undisclosed number of FIFA guests. The hospital did not encompass the provision of medical care for spectators or on-the-field emergency medical care, as these services were overseen by other health care providers within the country. Offered health services consisted of sports medicine, orthopedic surgery, physiotherapy, dentistry, general surgery, emergency medicine, internal medicine, cardiology, radiology, podiatry, and recovery services. All services were free of charge for the participating teams.

## Initial planning

Our initial challenge arose from the paucity of literature addressing imaging studies conducted during past World Cups, which made it difficult to formulate a well-informed strategy for radiological operations. Despite the existence of injury surveillance studies dating back to the 1998 World Cup [6–8], none of these references contained pertinent imaging data. One FIFA release from the 2018 World Cup in Russia mentioned 39 scans conducted, shedding limited light on the imaging aspect [9]. Notably, radiology data have previously been published for elite multisport events, such as the Olympic and Commonwealth Games [4, 10].

Although we acknowledge the limitations of comparing multimodality sports competitions and the FIFA World Cup, we chose to base our operational planning on these available data. Factoring in an estimated imaging utilization rate of approximately 10% of all athletes, we anticipated that with 832 athletes participating in the tournament, there would be a need for approximately 80 to 100 radiological examinations for athletes throughout the entire competition.

Considering the group stage’s schedule of 4 daily matches, which occurred at 1:00 p.m., 4:00 p.m., 7:00 p.m., and 10:00 p.m., we decided to maintain a round-the-clock 24/7 on-site radiology team throughout the tournament. This approach anticipated the likelihood of late-night athlete arrivals after the last match of the day. Although our regular patient volume experienced a decrease, we remained committed to offering radiology services to our regular public, particularly during the morning hours.

## Workflows in the World Cup

During the tournament, access to the hospital was enabled through a 24-h centralized call center denominated the Medical Command Center (MCC), which coordinated all medical services around the tournament. Staff from national teams requiring medical assistance contacted the MCC and were directed to the appropriate medical provider.

Radiology requests were simplified by allowing team physicians (who were licensed in the country for the duration of the tournament) to request any imaging modality directly with the radiology department, without the previous examination of the patient by an in-house physician. This approach significantly improved the process and was well received by team physicians.

An important factor in arranging the workflows was the aspect of privacy. Due to the nature of the competition and the use of one primary site for medical needs, it was important to ensure that each national team was able to access care without the knowledge of others, including athletes from other teams and normal hospital patients. This became one of the greatest considerations and impacts of the workflow design.

The scheduling of the needed exams was arranged by the MCC and the radiology team leader of the shift. Time requested by national team staff members was adhered to as closely as possible, while limiting service access to one team at a time whenever possible.

Upon arrival at the hospital, the patient and accompanying team staff members were greeted by an athlete relations staff member at the entrance. The athlete relations staff member communicated their arrival to the radiology team leader so that upon entering the radiology department, the team leader was awaiting the athlete at the reception desk and thus able to guide the athlete and their team, accompanying staff members throughout their visit to the department. Such communication was also necessary so that national teams did not cross paths with each other in their journey throughout the facility. The national team staff members were also requested to remain with the athlete as a cohesive group as a means of ensuring athletes’ complete privacy. A concise clinical history was obtained to inform the most suitable imaging protocol and to assist in imaging interpretation.

Upon completion of the examination, the national team staff members and athlete were able to have a direct consultation with the radiologist. Images were sent via a secure link to the nominated medical team members upon completion of an authorization form by the athlete. Communication between the radiology team leader and athlete relations staff was key to maintaining national team confidentiality throughout the visit; upon completion of their radiology visit, the athlete relations escorted the team members to the hospital exit and their awaiting transport.

## Radiology equipment in the World Cup

Compared to the Olympic Games, where imaging infrastructure is specifically set up for the tournament, we utilized our existing imaging structure, which is composed of two MRI magnets (1.5T and 3.0T), two US machines, one 64-slice CT scanner, and one digital system for radiography. The CT scanner features an intervention setup, with fluoro-CT capabilities and a video monitor integrated within the scanning room.

## Human resources in the World Cup

Technical and medical imaging expertise is paramount for the successful care of athletes during a major sports event and requires advanced planning. Technologists and radiologists typically engage in direct interactions with athletes, team delegations, physiotherapists, and physicians.

We leveraged our existing staff, who are experienced in sports-related imaging and interventions. This approach contrasts with the setup at the Olympic Games Polyclinic, where a dedicated facility is constructed exclusively for the games, and clinicians are either hired on a temporary basis or consist of volunteers. Our human resources involved with the competition consisted of 4 musculoskeletal radiologists, 13 technologists, 2 nurses, and 4 administrative staff. Remote neuroradiologists and emergency radiologists were available to discuss cases if needed, although their services ultimately went unused.

## Radiology examinations in the World Cup

Imaging services opened on a 24/7 basis 10 days before the start of the tournament and functioned up until 1 day after the final match.

Thirty-one out of the thirty-two national teams used our facility during the tournament. One national team decided not to use the centralized healthcare center facilities at all and used a private healthcare facility. Twenty-eight (28) national teams performed at least one imaging study, and 2 national teams utilized other services in our hospital; however, they did not request any imaging studies.

One hundred eighty (180) radiologic examinations and interventional procedures were performed. Of this total, 143 (79.4%) examinations were performed on 94 athletes, with the remaining 37 (20.6%) examinations and procedures performed on 23 FIFA staff, guests, referees, and team members. Most radiologic examinations consisted of MRI (65%), followed by radiograph (15%), US (9%), CT (3.5%), and image-guided procedures (6.5%) (Table 1). Radiology exhibited the highest demand among hospital services during the World Cup, representing 51.7% of all patient encounters.

**Table 1:** Total number of imaging modalities

Imaging modality	Total
MRI	117 (65)
XR	28 (15)
US	16 (9)
CT	6 (3.5)
CT-guided injection	1 (0.5)
US-guided injection	12 (6.5)
All	180

Data in parentheses are percentages

*MRI* magnetic resonance imaging, *XR* radiography, *US* ultrasound, *CT* computed tomography

**Fig. 1:**
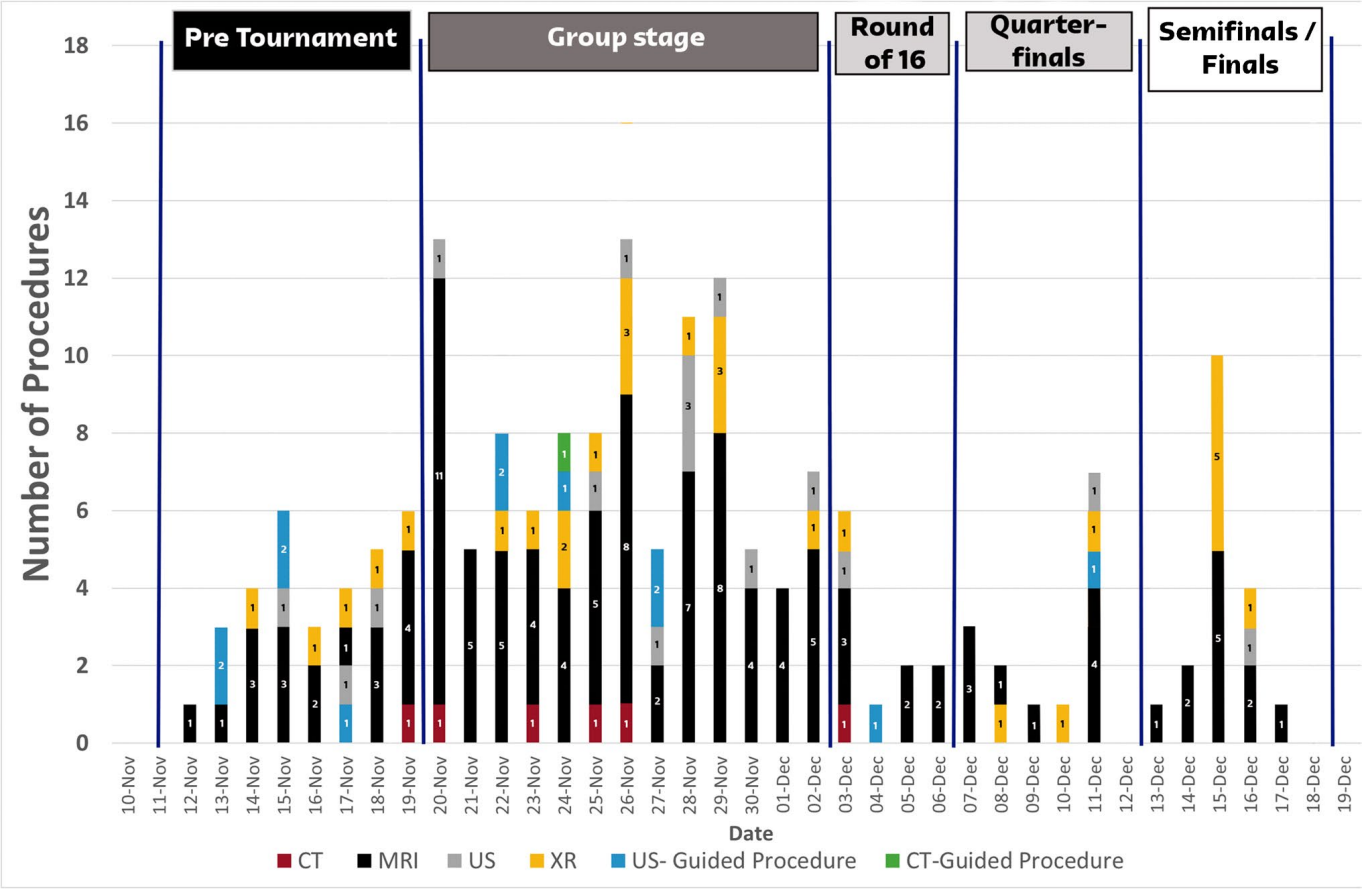
Distribution of imaging studies throughout the duration of the 2022 FIFA World Cup

**Fig. 2:**
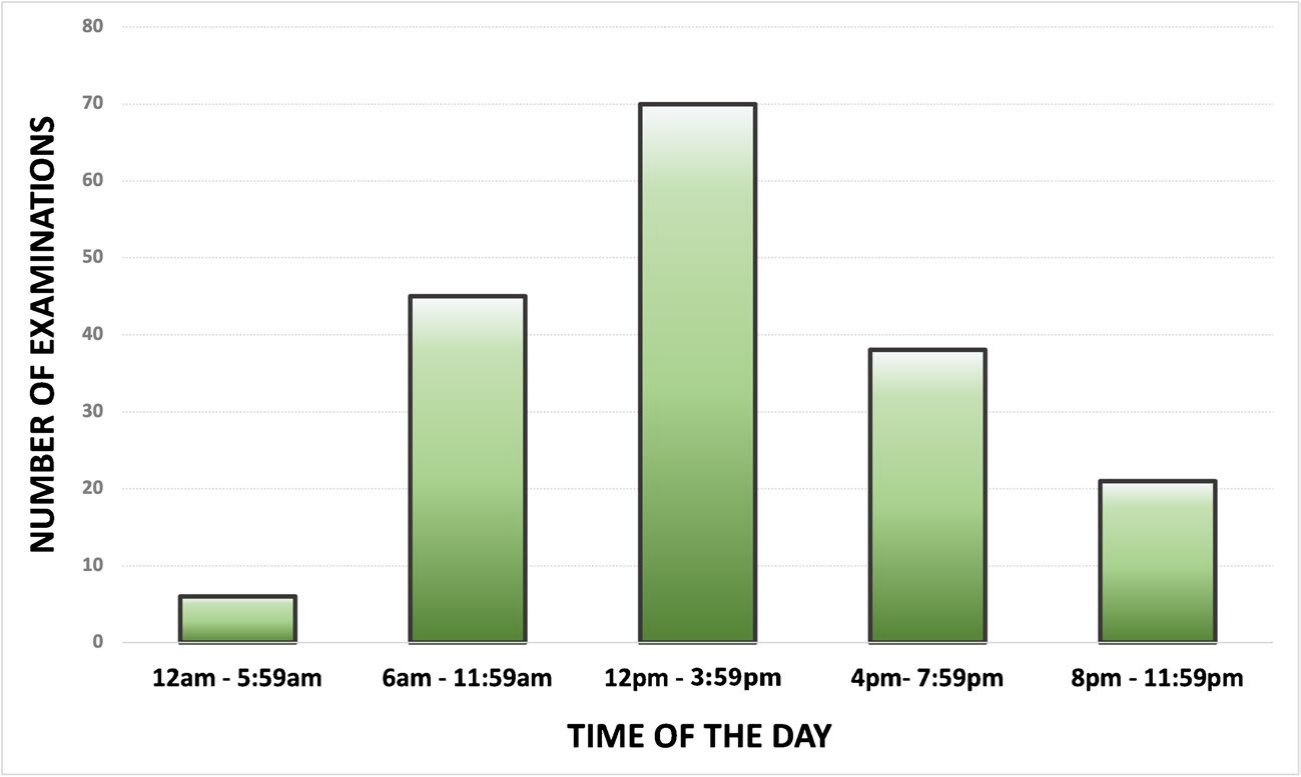
Distribution of examination times

Most imaging examinations were performed during the group stage of the tournament, with a peak around the second match of each team. A second peak of interest emerged during the semifinals/finals stage, which was related to the FIFA Legends Tournament, an ancillary competition organized by FIFA and featuring former international football players who have retired (Fig. 1). The busiest times in the radiology department were between 12 p.m. and 4 p.m. (40%), followed by the mornings from 6 a.m. to 12 p.m. (25%), and evenings from 4 p.m. to 8 p.m. (21.6%). Only 3.3% of the examinations were performed between 12 a.m. and 8 a.m. Figure 2 shows the distribution of examination times in the radiology department.

## Communication in the World Cup

Effective communication is a cornerstone in delivering accurate and high-quality radiology services; however, it is often overlooked within the management of radiology departments. To improve the groundwork for efficient communication, appropriate planning should commence at the organizational level.

Some key components pertinent to efficient interprofessional communication in radiology care for an international sports event encompass overcoming language barriers, fostering trust, and ensuring the rapid delivery of radiology reports and images. To address these challenges, certain national teams have integrated radiologists into their medical staff. From an organizational perspective, a primary objective of the World Cup radiology department was to offer each national team a seamless service consisting of the facilities they would typically have in their home country.

Language barriers have a major impact on the quality of health care and usually occur when individuals do not share a native language. Language barriers can often result in challenges related to comprehending radiology reports, ultimately diminishing the reliability of the information provided. English is the preferred language in a global health care setting and is the most spoken language in the world, including native and nonnative speakers [11]. However, nonnative English speakers may face difficulties in scientific and medical communication [12]. To overcome language barriers and considering that our in-house radiologists were fluent in several languages, we provided radiology discussions and reports in English, Spanish, Portuguese, French, and Arabic, depending on the national teams’ preferences. In total, 62% of discussions and consultations were provided in English, 16% were provided in French, 12% were provided in Spanish, 9% were provided in Portuguese, and 1% were provided in Arabic.

Trust is the ability to rely on another person in situations of uncertainty. It is present in the context of interprofessional relationships and is more difficult to measure than efficiency or productivity [13]. During international events, team physicians and in-house radiologists typically lack a preexisting professional relationship. Consequently, the foundation of trust is often nonexistent due to unfamiliarity among these professionals. One of the main goals for radiology care in the World Cup was to establish trust among all team physicians and athletes.

Following imaging acquisition, consultations took place in the radiologist’s office, where the in-house radiologist, team health care professional, and athlete collectively reviewed the images for each case.

The improvement of radiology reports with key images and hyperlinks was explored to provide a clearer demonstration of findings. In addition, we provided a supplemental audiovisual report, where the radiologist created a short video with an explanation of findings, aiming to improve the referring provider’s overall experience and understanding of the imaging results [14–16]. An audiovisual reporting tool (AVR digital, AVR Software Development for Healthcare, Sao Paulo, Brazil) was available inside the diagnostic viewer, allowing the simultaneous capture of the screen content, the radiologist’s voice, and the mouse cursor. The final audiovisual report was incorporated within the picture archiving and communication system (PACS) as a new series. Video 1 is an example of an audiovisual report of a chronic groin injury (available in the online version).

**Fig. 3:**
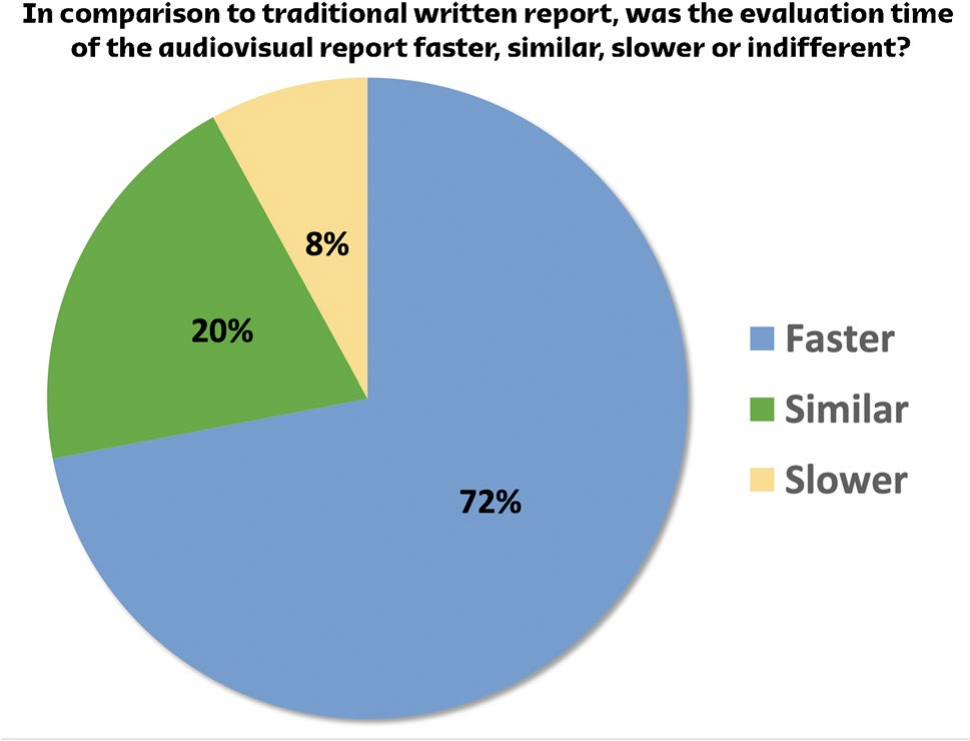
Distribution of answers given to question: “In comparison to traditional written report, was the evaluation time of the audiovisual report faster, similar, slower or indifferent?”

**Fig. 4:**
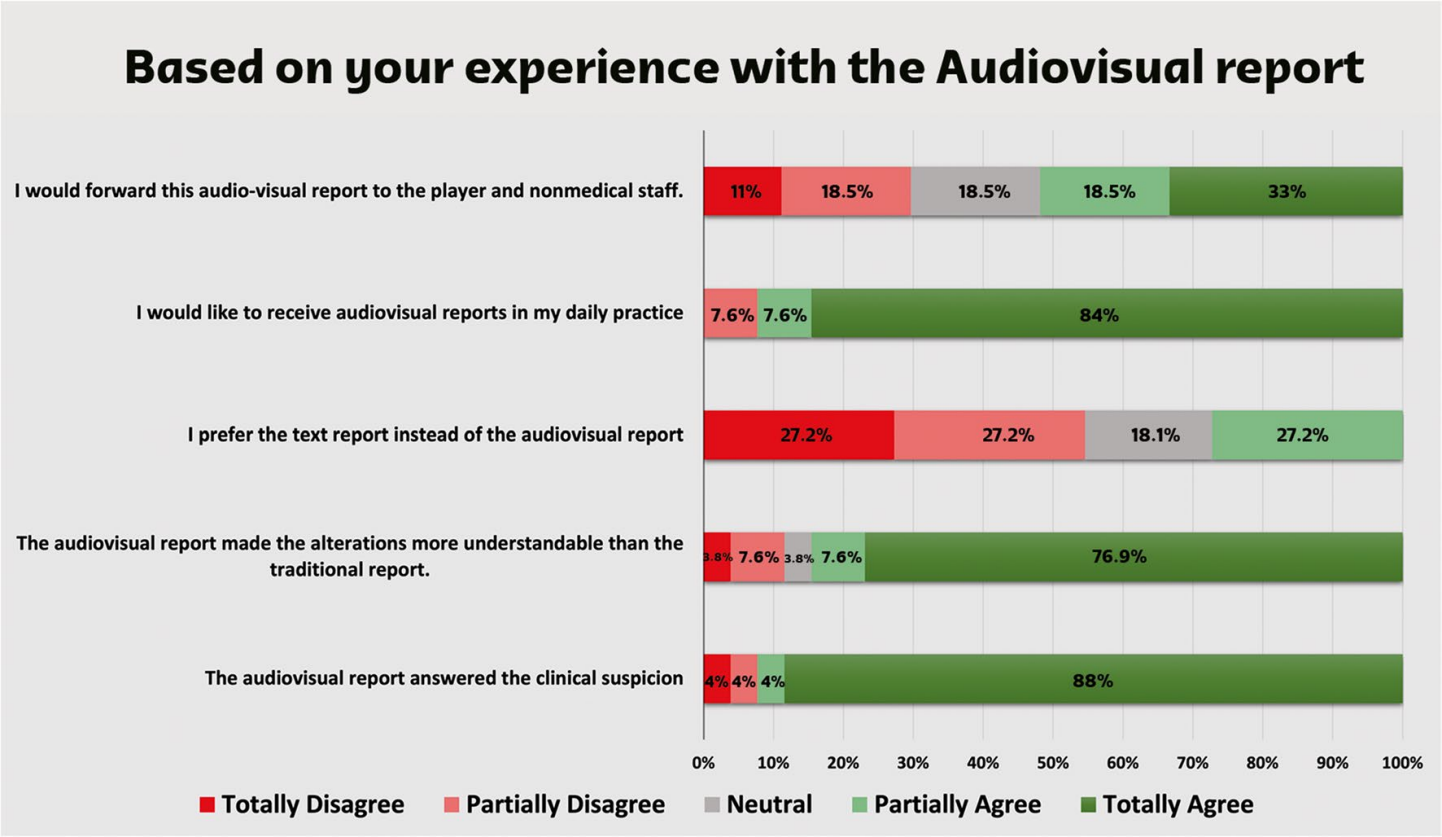
Distribution of answers given to the questions regarding providers’ experiences with the audiovisual report

To assess referral physicians’ opinions on the audiovisual reports and obtain feedback for improvement, a survey consisting of 7 questions was sent through a link to 43 health care professionals from 27 national teams who received audiovisual reports during the competition. Twenty-five (25) professionals answered the survey, for a response rate of 58.1%. Most responders were orthopedic surgeons (44%), followed by sports medicine physicians (36%), physiotherapists (16%), and general practitioners (4%). Based on the responses obtained through the questionnaire (Figs. 3 and 4), we found that the implementation of the audiovisual report had a positive impact on communication between the imaging department and referring providers. This improvement contributed to enhancing the comprehension of imaging studies. Interestingly, it was observed that only 51.5% of the referring providers expressed a willingness to disseminate the audiovisual report to both players and nonmedical staff. This was also observed in a prior study and might be related to unfamiliarity about how this innovative report format could affect the athlete’s comprehension of their own case [14].

All radiology images and reports were transmitted to team physicians via the image sharing portal (Carestream, Philips Healthcare, The Netherlands). Additionally, when requested, we forwarded the images to other health care professionals.

## Recommendations

Diagnostic imaging played a central role in the multidisciplinary health care approach used in the 2022 FIFA Football World Cup. Based on our experience, the careful planning of equipment, technology requirements, workflows, and service scheduling is imperative. Our primary strategies included flexibility, streamlining imaging requests, and fostering effective communication. In particular, we undertook the following key actions:

1. Overcoming language barriers by offering consultations and radiology reports in multiple languages.

2. Establishing trust through radiology consultations and promoting alternative reporting formats, with a focus on audiovisual reports.

3. Expediting the rapid online delivery of radiology reports and images.

A centralized approach to radiology care is unlikely to be replicated in future football World Cups, given the geographical and decentralized nature of upcoming events. Nevertheless, our experience offers comprehensive insight into radiology services and provides valuable information for prospective event organizers and radiology stakeholders. These data can be useful in resource allocation, thereby enabling the improvement and optimization of radiological services for forthcoming international professional football events.

## Supplementary information

ESM 1 (MP4 5387 kb)
